# Enhancer Tuning by Sequence-Specific Repressors

**DOI:** 10.64898/2026.09.11.751074

**Published:** 2026-09-14

**Authors:** Lucia Ichino, Kaelan J. Brennan, Benjamin J. Mallory, Tomek Swigut, Stephanie C. Bohaczuk, Jaaved Mohammed, Devaraja G. Mudeppa, Saman Tabatabaee, Chunyang Ni, Andrew B. Stergachis, Joanna Wysocka

## Abstract

Enhancers integrate combinatorial inputs from sequence-specific transcription factors (TFs) and their activity must be calibrated to achieve precise spatiotemporal control of transcript dosage. Here we demonstrate that the sequence-specific repressors SNAI1 and SNAI2 ( *i.e.* SNAIL and SLUG) quantitatively tune enhancer activity. In human neural crest cells, SNAI1/2 occupy a subset of active enhancers, where their depletion increases H3K27ac, chromatin accessibility, and enhancer regulatory potential. Changes in SNAI1/2 binding motifs contribute to enhancer divergence between human and chimpanzee, suggesting an evolutionary role for the repressor-mediated tuning. Single-molecule chromatin profiling using Deaminase-Assisted Fiber-seq (DAF-seq) reveals that individual enhancers toggle between an ensemble of open and nucleosome-dense chromatin states. While transcriptional activators increase the fraction of the open states, SNAI1/2 shift the equilibrium toward nucleosome-occupied states. This impedes binding of activator TFs, without fully repressing the enhancer. We propose that SNAI1/2 function as a molecular dimmer switch—modulating nucleosome dynamics to calibrate enhancer output.

## Full Text Availability

The license terms selected by the author(s) for this preprint version do not permit archiving in PMC. The full text is available from the preprint server.

